# Three-Level Concavity Apical Pediculectomy During Intraoperative Neuromonitoring Loss: Following the Checklist Resulted in an Ambulatory Patient

**DOI:** 10.7759/cureus.64727

**Published:** 2024-07-17

**Authors:** Danner W Butler, Tyler C McDonald, Sudhir Suggala, Richard Menger

**Affiliations:** 1 Neurosurgery, University of South Alabama, Mobile, USA; 2 Pediatric Orthopedics, University of South Alabama, Mobile, USA; 3 Neurological Surgery, USA Health Physicians Group/University of South Alabama College of Medicine, Mobile, USA

**Keywords:** neuro-surgery, complex spine deformities, pediculectomy, neuromonitoring loss, : adolescent idiopathic scoliosis

## Abstract

Adolescent idiopathic scoliosis is the most common form of scoliosis, with severe cases leading to a decline in patients with worsening angulation of deformity. Technical nuances of spinal flexibility and cord type based on the extent of the deformity may impact operating safety and outcome, with risks including neurological loss during and after surgical intervention. Here we present a case of posterior osteotomy and correction of a patient with adolescent idiopathic scoliosis with a T2 - L3 fusion in which transcranial motor evoked potentials (TcMEPs) and somatosensory evoked potentials (SSEPs) were lost intraoperatively, thus requiring application of operative consensus guidelines for the loss of neuromonitoring data. Particularly, the discussion focuses on the decision-making process that resulted in the complete recovery of TcMEPs and SSEPs post-operatively.

## Introduction

Adolescent idiopathic scoliosis (AIS) is the most common form of scoliosis, affecting between 1-3% of the population [1]. Defined as a spinal curvature of greater than 10 degrees, AIS consists of a coronal, sagittal, and rotational plane deviation [1]. Beyond 50 degrees of curvature, AIS defects can result in major adverse health conditions such as debilitating back pain and body dysmorphia and may even progress to decreased pulmonary function once curvature has reached 90 degrees [2-4]. While orthotic management may suffice between 25-50-degree deficits, surgical intervention is recommended once curvature has increased beyond a 50-degree angle. Technical nuances of flexibility, spinal cord type, and lung or cardiac function may impact operating safety and overall outcome [2-5]. Complications, including neurological loss, must additionally be considered, both pre-operatively and intraoperatively during the decision-making process [6-7]. 

Additionally, increasing deformity angle ratios (DAR), or curve magnitude per level of spinal deformity, increases risks of intraoperative neuromonitoring loss, thus necessitating further pre-operative consideration for higher DARs [4]. Recent guidelines established by Vitale et al. and more recently, Lenke et al, have provided algorithmic methodology in the surgical management of both unstable and stable complex spine cases [8, 9]. Such guidelines were established by groups of expert spine surgeons, with a focus on creating a team approach to crisis situations. While guidelines are established, clinical case reports of the effectiveness of such decision-making processes remain limited.

Here we present a case of posterior osteotomy and correction of a patient with AIS in which transcranial motor evoked potentials (TcMEPs) and somatosensory evoked potentials (SSEPs) were lost intraoperatively. Discussion focuses on the stepwise decision-making process and application of consensus guidelines for surgeon decisions during neuromonitoring changes, as established by Vitale et al. and more recently, Lenke et al [8, 9]. The patient had complete recovery of TcMEPs and SSEPs post-operatively. We explore the technical lessons learned from this experience.

## Case presentation

**Figure 1:**
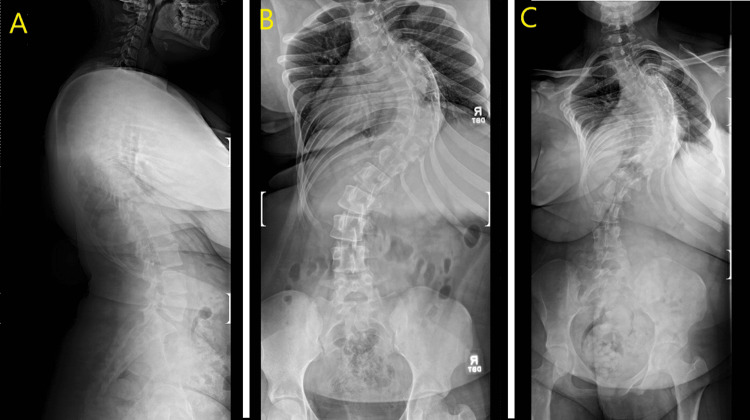
Pre-operative standing X-ray showing A) sagittal view, B) anteroposterior (AP) view, and C) posteroanterior (PA) view of spinal deformity

A 13-year-old female originally presented in 2019 with evidence of scoliosis showing approximately 100 degrees of curvature, improving to 90 degrees with traction film. The patient re-presented in 2023 with worsening back pain. Physical exam revealed bilateral strength 5/5 in the upper and lower extremities, as well as sensation intact to bilateral lower extremities globally (Figure 1).

**Figure 2:**
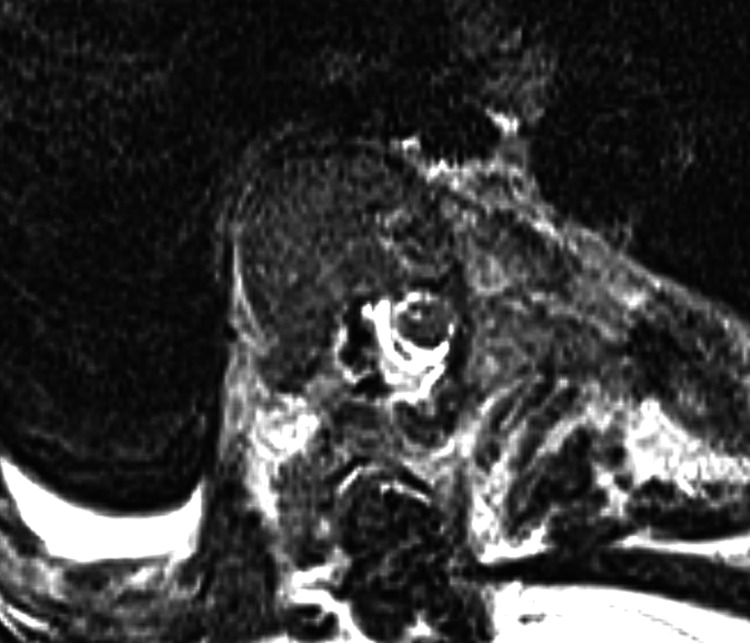
Axial MRI T2 imaging showing a Type 3 spinal cord at the apex.

However, MRI revealed a type 3 spinal cord as well as a small right pleural effusion, concerning for a diagnosis of AIS affecting pulmonary status (Figure 2) [7]. 

Her deformity angular ratio (DAR) was 12.25 in the coronal plane, 8.875 in the sagittal plane, and 21.125 overall [4]. Subsequently, she was indicated for complex spine reconstruction through the pediatric spine complex care pathway. This is a two-attending model with orthopedic surgery and neurosurgery. Patient consent was obtained. IRB approval was not required for this single-patient case report.

A T2 - L3 incision was performed under 15 lb traction. Upon completion of facetectomy, posterior column osteotomies were planned. The T10 - T11 osteotomy was performed without any unexpected events. Subsequently, during the T9 - T10 osteotomy, left lower extremity TcMEPs met alert criteria to complete loss furthered by unilateral complete loss of left-sided SSEPs.

The best-practices guidelines were applied for the loss of neuromonitoring [9]. An immediate increase of mean arterial pressure (MAP) was performed via closed-loop communication with the senior anesthesia provider. Upon releasing traction, we looked to shorten the spinal cord and protect against any drift against the medial pedicle. We elected to place a rod on the right side of the periapical levels to enact compression on the convex (right) side. We then placed pedicle screws at T7 and T10 via the freehand method on the convex side before connecting a titanium rod between the two and compressing these levels. This afforded some slight improvement in neuromonitoring signals, but signals were still concerning with no large improvement. After a half hour, with proper MAP maintenance showing no improvement, we made the decision to give Solumedrol at 30 mg/kg for the next hour.

According to the guidelines, an outside surgeon, in this case a third surgeon, was called for finite decision-making. This confirmed our decision matrix of either contusion from PCO (posterior column osteotomy) or spinal cord shift. The wake-up test would not change immediate management and a pedilectomy was indicated. 

The T7, T8, and T9 periapical pedicles on the concave side were removed before the lamina on the left side. The pedicles were carefully resected while protecting the underlying spinal cord and nerve root. The left/ventral aspect of the pedicles/ventral wall was removed as well. There was visual kinking at T7 - T9, and upon release, the spinal cord was seen to show pulsations. The SSEPs returned to above alert level, and the left psoas and abdomen/foot TcMEP signal returned as well. At this point, we decided to place a provisional fixation and end the case. We placed pedicle screws at T4, T5, L1, and L2 on the right side before a 5.5mm titanium rod was cut and bent to match the in-situ positioning and placed into these pedicle screws. The immediate post-operative wake-up test was full strength.

**Figure 3:**
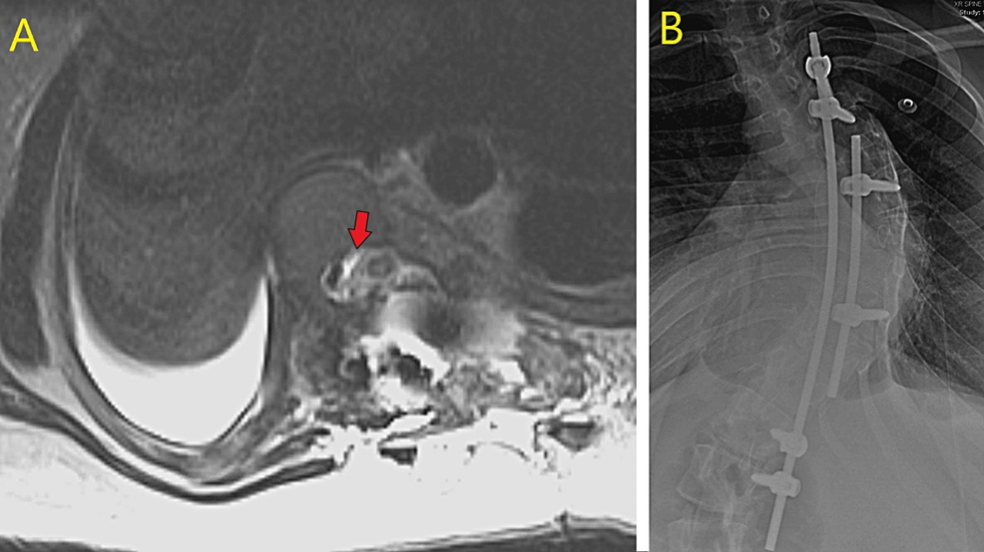
(A) Axial T2 hyperintensity at the cord apex status post pediculectomy; (B) Intraoperative temporary fixation.

The patient was monitored inpatient for one week with no neuromuscular deficits noted. MRI was performed post-operatively showing a T2 hyperintensity at the apex of the spinal cord at T8 (Figure 3).

**Figure 4:**
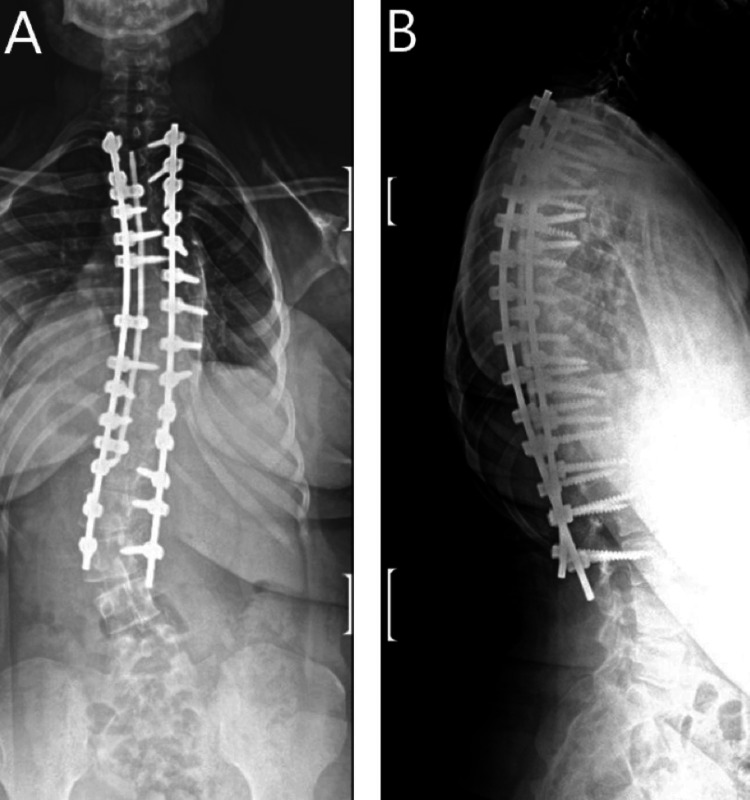
Anteroposterior (A) and lateral (B) post-operative X-ray showing T2 - L3 fusion.

With reassurance of stable neurological function, in consultation with other colleagues, the surgical wound was reopened, and proper instrumentation was placed for T2 - L3 fusion. At three month follow-up, the patient reported markedly reduced back pain with 5/5 strength bilaterally. Imaging revealed stable instrumentation with alignment well maintained (Figure 4).

## Discussion

Observations

There were several preoperative warning criteria in this case that resulted in a high risk of complication. The first is the high deformity angular ratio (DAR), as the patient had a curve over 100 degrees. A combined DAR of over 20 has a substantially higher risk of neuro-monitoring loss [4]. Additionally, the patient had a type 3 cord compression, defined as when no cerebrospinal fluid (CSF) is any longer visible around the spinal cord on MRI [10]. This contrasts with a type 1 cord, which only involves an epidural extension, and type 2, in which CSF is still visible around the cord 7. A type-3 compression has a 28 times higher risk of having an intraoperative neuromonitoring loss [5]. Lastly, unlike typical AIS, there was also a kyphotic component to the deformity, of which surgical correction may stretch the anterior-based watershed vasculature.

Application of best practice guidelines

Recent clinical guidelines have established the necessity of organized, team-based thinking in cases of intraoperative neuromonitoring loss (IONM) in both the stable and unstable spine. Vitale et al established a step-by-step process in IONM changes in the stable spine, highlighting the need to (1) gain control of the room as the surgeon, (2) address anesthetics (optimize MAP, hematocrit, pH and partial pressure of carbon dioxide (pCO2), consider wake-up test), (3) assure proper technical/neuro-physiological setup, and (4) address surgical needs going forward [8]. Furthermore, and more specifically in our case, Lenke et al. expanded on the unstable spine, addressing the need for similar stepwise, team-based thinking [9]. Our specific case qualified as a Stage 2a alert, as destabilization occurred after posterior column osteotomy. Subsequently, we followed the outlines defined by Lenke et al., opting to 1) remove traction, 2) stabilize with temporary rods, and 3) assess for dural compression and perform necessary pediculectomy for relief [9]. Descending neurogenic-evoked potentials were not available to assess further levels of dysfunction in our case. This “stepwise” level of thinking was consistently followed, eventually leading to a full recovery for our patient.

Lessons

Instrumentation should be placed before posterior column osteotomies on the convexity side above and below the main apex prior to osteotomy. A temporary rod can be placed to stabilize as any type of compression will shorten the spine. This may reduce the technical difficulty of a concavity PCO as well as protect against any shift migration. These are the two most likely causes of intraoperative neuromonitoring loss.

## Conclusions

In the operating room, cases may not go as planned, leading to the need for stepwise decision-making to ensure further crisis is averted. This case report highlights such an instance, providing evidence of the effectiveness of adhering to the suggested guidelines established to handle complex spinal deformity cases with a level mind. In addition, the case is an example of the team-based practices that are necessary to handle crisis situations within the operating room. Timing of instrumentation and posterior column osteotomies are an additional surgical lesson of the reported case. Continued reflection upon such difficult cases allows for the betterment of patient care and surgical performance in the future.
